# Post-acute symptom persistence following SARS-CoV-2, RSV, and influenza in children: A retrospective cohort study

**DOI:** 10.1097/MD.0000000000050497

**Published:** 2026-09-11

**Authors:** Firat Bedir, Velit Demir

**Affiliations:** a Department of Pediatrics, Istanbul Medipol University Hospital, Istanbul, Turkey.

**Keywords:** influenza, long COVID, pediatric, post-acute symptoms, postinfectious morbidity, SARS-CoV-2, viral respiratory infections

## Abstract

Persistent symptoms following acute viral respiratory infections are increasingly recognized in children. While post-acute sequelae of severe acute respiratory syndrome coronavirus 2 (SARS-CoV-2; long COVID) have been extensively studied, whether comparable post-acute morbidity occurs after other major respiratory viruses and whether clinical patterns differ across pathogens remains incompletely characterized. This retrospective cohort study included children aged 0 to 18 years with reverse transcription polymerase chain reaction–confirmed SARS-CoV-2 (n = 1540), respiratory syncytial virus (RSV; n = 2150), or influenza (n = 2050) infections evaluated between January 2020 and December 2024 at a tertiary university hospital system in Istanbul, Turkey. Persistent symptoms were assessed at clinician-documented follow-up visits 4 to 6 weeks after virological diagnosis. Multivariable logistic regression identified independent predictors for each pathogen. Bonferroni correction was applied for multiple comparisons. Model calibration was assessed using the Hosmer-Lemeshow test, and discriminative ability by the area under the receiver operating characteristic curve. Among 5740 children, persistent symptoms at 4 to 6 weeks were documented in 24.0% (95% confidence interval [CI] = 22.2–25.8) with RSV, 22.0% (95% CI = 19.9–24.1) with SARS-CoV-2, and 21.2% (95% CI = 19.4–23.0) with influenza (*P* = .086). While overall post-acute prevalence was comparable across the 3 viruses, symptom profiles differed markedly: RSV-related post-acute morbidity was dominated by respiratory and feeding difficulties in young infants; SARS-CoV-2 predominantly produced neurocognitive and systemic complaints in school-aged children; and influenza was characterized by sustained systemic symptoms. Hypoxemia was the strongest predictor for RSV (adjusted odds ratio [aOR] = 2.4, 95% CI = 1.7–3.3), while age >6 years (aOR = 2.1, 95% CI = 1.5–2.9) and hospitalization (aOR = 1.9, 95% CI = 1.4–2.6) were leading predictors for SARS-CoV-2. Age-stratified analyses demonstrated a significant virus × age interaction (*P* < .001), with the highest prevalence observed after RSV among infants and after SARS-CoV-2 among school-aged children. Hosmer-Lemeshow testing confirmed adequate calibration for all models (all *P* > .05). Inverse probability weighting sensitivity analysis yielded consistent results. Post-acute symptom persistence following pediatric viral respiratory infections is common and follows virus-specific patterns. Overall post-acute symptom prevalence was comparable across the 3 viruses, but symptom profiles were strikingly virus-specific and age-dependent. This direct comparison of 3 major respiratory viruses demonstrates that post-acute morbidity extends beyond SARS-CoV-2 and varies by pathogen and age. These findings support the development of pathogen-informed follow-up considerations.

## 1. Introduction

Acute viral respiratory infections are among the leading causes of illness, emergency department visits, and hospitalizations in children worldwide.^[1–3]^ In the post-pandemic era, severe acute respiratory syndrome coronavirus 2 (SARS-CoV-2), respiratory syncytial virus (RSV), and influenza remain the 3 dominant viral etiologies responsible for a substantial proportion of pediatric respiratory morbidity.

Post-acute symptoms following pediatric SARS-CoV-2 infection – often referred to as long COVID – have been extensively documented. Meta-analytic data from 40 studies estimate a pooled prevalence of approximately 23.4% (95% confidence interval [CI] = 15.8%–32.5%) in children,^[4–6]^ with fatigue, headache, sleep disturbance, and concentration difficulties among the most commonly reported complaints.^[7,8]^ Prospective cohort studies have identified acute illness severity, older age, and preexisting conditions as consistent risk factors.^[9–11]^ However, comparative data examining whether similar post-acute symptom persistence occurs following other major pediatric respiratory viruses remain limited.^[12]^

Emerging evidence suggests that RSV bronchiolitis may produce prolonged respiratory morbidity beyond the acute illness phase. Post-discharge studies have documented persistent cough, wheezing, and feeding difficulties lasting weeks to months after hospitalization,^[13,14]^ and long-term follow-up data link severe RSV infection to subsequent recurrent wheezing and asthma development.^[15,16]^ Similarly, influenza has been associated with postinfectious fatigue, myalgia, and functional impairment extending beyond the typical resolution window.^[17,18]^ Nevertheless, whether these post-acute manifestations are comparable in prevalence to post-SARS-CoV-2 sequelae, and whether they possess distinct risk factor profiles, has not been systematically evaluated within a unified clinical framework.

Most existing studies focus on a single viral etiology, limiting direct comparative analysis. Although experienced clinicians intuitively tailor follow-up intensity based on acute illness characteristics, evidence-based virus-specific recommendations for post-acute monitoring remain lacking.^[19–21]^ A unified comparative analysis encompassing the 3 predominant pediatric respiratory viruses within the same institutional setting and time frame could bridge this gap.

Accordingly, this study aimed to compare post-acute symptom persistence at 4 to 6 weeks following pediatric SARS-CoV-2, RSV, and influenza infections within a unified real-world clinical cohort and to identify virus-specific independent predictors of symptom persistence to inform risk-stratified follow-up strategies.

## 2. Materials and methods

### 2.1. Study design and setting

This retrospective cohort study was conducted within the Istanbul Medipol University hospital system, which includes 2 tertiary-care university hospitals operating under the same institutional administration, electronic medical record (EMR) infrastructure, and clinical protocols. Both facilities serve as referral centers with dedicated pediatric emergency departments, inpatient wards, and subspecialty outpatient clinics. Consecutive children aged 0 to 18 years with reverse transcription polymerase chain reaction (RT-PCR)–confirmed SARS-CoV-2, RSV, or influenza infections evaluated between January 2020 and December 2024 were identified through the institutional EMR. The study was approved by the Istanbul Medipol University Non-Interventional Ethics Committee (Decision No: 1460, Date: 27.11.2025). As this was a retrospective analysis of existing clinical records, ethics committee approval was obtained prior to data extraction, analysis, and manuscript preparation; the approval date therefore postdates the clinical data-collection period (2020–2024) by design, consistent with standard practice for retrospective observational research. Informed consent was waived due to the retrospective, anonymized nature of data collection. This study followed the Strengthening the Reporting of Observational Studies in Epidemiology guidelines.

### 2.2. Virological confirmation

All infections were confirmed by real-time RT-PCR performed on nasopharyngeal swab specimens. SARS-CoV-2 testing used Bio-Speedy SARS-CoV-2 RT-qPCR (Bioeksen); RSV and influenza (A/B) testing used the Allplex Respiratory Panel (Seegene). Results were available within 4 to 12 hours.

### 2.3. Inclusion and exclusion criteria

Inclusion criteria were age 0 to 18 years; RT-PCR–confirmed SARS-CoV-2, RSV, or influenza infection; and documented outpatient follow-up visit at 4 to 6 weeks after virological diagnosis. Only the first confirmed episode per patient per virus type was included.

Exclusion criteria were co-infection with 2 or more study viruses; chronic respiratory disease (e.g., cystic fibrosis, bronchopulmonary dysplasia, primary ciliary dyskinesia); immunodeficiency (primary or secondary); documented preexisting symptoms identical to those assessed as post-acute outcomes; receipt of immunomodulatory therapy within 30 days before infection; or incomplete core data.

### 2.4. Patient selection and flow

EMRs were queried for all children with positive RT-PCR results during the study period. The initial query yielded 8246 unique patient records. After applying exclusion criteria, 5740 were included: 1540 with SARS-CoV-2, 2150 with RSV, and 2050 with influenza. The most common reasons for exclusion were absence of a documented follow-up visit (n = 1287; 52% of exclusions), co-infection (n = 534; 21%), and chronic respiratory disease (n = 412; 16%).

### 2.5. Data collection

Demographic characteristics and acute clinical features were extracted from EMRs by 2 investigators (FB and VD) independently using a standardized data abstraction form. Discrepancies were resolved by consensus review with reexamination of source records.

### 2.6. Definitions

Post-acute symptoms were defined as clinical complaints documented at the 4- to 6-week follow-up visit not present prior to the index infection or representing clinically meaningful worsening. The window was measured from the date of virological confirmation to the follow-up visit date. The acute phase was defined as the period from symptom onset through resolution of primary symptoms (defervescence and clinical stabilization), typically 7 to 14 days. Fever was defined as axillary temperature ≥38.0°C by digital thermometer; low-grade fever as 37.5 to 37.9°C; prolonged fever as fever persisting or recurring beyond 7 days after symptom onset. Hypoxemia was defined as peripheral oxygen saturation <92% on pulse oximetry at any point during acute illness. Prematurity was defined as birth before 37 completed weeks of gestational age. Elevated inflammatory markers were defined as C-reactive protein >20 mg/L or procalcitonin >0.5 ng/mL (highest value during acute illness). Fatigue in infants was assessed as reduced activity, decreased alertness, or prolonged sleep beyond pre-illness baseline (caregiver report + clinical observation). Feeding difficulties were defined as oral intake <50% of pre-illness baseline or requirement for modified feeding techniques.

### 2.7. Symptom classification

Post-acute symptoms were categorized into 4 domains: systemic (fatigue, prolonged fever, myalgia); neurocognitive (headache, concentration difficulties, sleep disturbance); respiratory (persistent cough, wheezing, increased work of breathing); and feeding-related (feeding difficulties in infants).

### 2.8. Outcome definition

The primary outcome was the presence of any persistent symptom at 4 to 6 weeks, coded as binary (present/absent). Persistent symptoms were identified from structured clinician documentation in the EMR and were retrospectively extracted using a standardized data abstraction form. No validated patient- or caregiver-reported questionnaire, standardized symptom severity scale, or long-COVID-specific instrument (e.g., the International Severe Acute Respiratory and Emerging Infection Consortium pediatric follow-up questionnaire) was administered during routine follow-up. Consequently, the absence of a documented symptom could not always be distinguished from the absence of systematic inquiry into that symptom domain; this limitation is discussed further below.

### 2.9. Statistical analysis

Categorical variables were summarized as frequencies and percentages; continuous variables as medians with interquartile ranges. Baseline characteristics were compared using chi-square tests for categorical variables and Kruskal–Wallis tests for continuous variables. Post-acute symptom prevalence was compared across viral groups using chi-square tests with Bonferroni correction for pairwise comparisons (adjusted significance threshold *P* < .017 for 3 pairwise comparisons).

Multivariable logistic regression models were constructed separately for each viral cohort. Candidate predictors were selected based on clinical relevance and univariable screening (*P* < .10). All models were adjusted for age, sex, and hospitalization status. Variance inflation factors were calculated to assess multicollinearity; all variance inflation factor values were <2.5, indicating no problematic collinearity. Model calibration was assessed using the Hosmer-Lemeshow goodness-of-fit test, and discriminative ability was quantified using the area under the receiver operating characteristic curve (AUC/C-statistic). Results are reported as adjusted odds ratios (aOR) with 95% CIs.

Post hoc power analysis was performed to characterize the precision of the fixed, predetermined retrospective sample rather than to justify sample size a priori, and confirmed >95% statistical power to detect a 5-percentage-point difference in post-acute symptom prevalence between any 2 viral groups at α = 0.05, given the minimum group size of 1540 patients. Consistent with current recommendations, primary emphasis throughout the Results and Discussion is placed on effect sizes and 95% CIs rather than on *P* values alone.

### 2.10. Missing data handling

Patients with missing follow-up data were excluded from the primary analysis (complete-case analysis). All included patients had a documented 4- to 6-week follow-up visit, as required by the inclusion criteria; however, a subset had incomplete covariate data required for the multivariable regression models, and complete-case analysis was therefore used for those models (Table 1). To evaluate potential selection bias, baseline characteristics of included versus excluded patients were compared using standardized mean differences (SMDs); an SMD threshold of <0.10 was considered indicative of negligible imbalance. In addition, a sensitivity analysis using inverse probability weighting (IPW) was performed to assess the robustness of the primary findings to potential informative missingness.

**Table 1:** Baseline characteristics of the study population by viral etiology.

Characteristic	SARS-CoV-2 (n = 1540)	RSV (n = 2150)	Influenza (n = 2050)	*P* value
Median age (IQR)	7.4 yr (4.2–11.8)	10.5 mo (4–18)	7.2 yr (3.9–12.1)	<.001
Age <6 mo	92 (6.0%)	1204 (56.0%)	164 (8.0%)	<.001
Age 6–12 mo	139 (9.0%)	624 (29.0%)	205 (10.0%)	
Age 13 mo–6 yr	570 (37.0%)	215 (10.0%)	738 (36.0%)	
Age >6 yr	739 (48.0%)	107 (5.0%)	943 (46.0%)	
Male sex, n (%)	801 (52.0%)	1118 (52.0%)	1128 (55.0%)	.11
Hospitalization, n (%)	477 (31.0%)	774 (36.0%)	595 (29.0%)	.002
Hypoxemia*, n (%)	123 (8.0%)	624 (29.0%)	41 (2.0%)	<.001
Prematurity†, n (%)	108 (7.0%)	237 (11.0%)	123 (6.0%)	<.001
Palivizumab, n (%)	–	65 (3.0%)	–	–
Elevated CRP‡, n (%)	231 (15.0%)	301 (14.0%)	369 (18.0%)	.004
Missing follow-up, n (%)	231 (15.0%)	258 (12.0%)	267 (13.0%)	.06

Data are presented as n (%) or median (IQR). *P* values from chi-square test (categorical) or Kruskal–Wallis test (continuous).

CRP = C-reactive protein, IQR = interquartile range, RSV = respiratory syncytial virus, SARS-CoV-2 = severe acute respiratory syndrome coronavirus 2, SpO_2_ = peripheral oxygen saturation.

* SpO_2_ < 92% on pulse oximetry at any point during acute illness.

† Birth before 37 completed weeks of gestational age.

‡ CRP > 20 mg/L or procalcitonin > 0.5 ng/mL.

All analyses were performed using IBM SPSS Statistics version 28.0 (IBM Corp.) and R version 4.3.1 (R Foundation for Statistical Computing). Two-sided *P* < .05 was considered statistically significant, with Bonferroni-adjusted thresholds for pairwise comparisons.

## 3. Results

### 3.1. Study population

A total of 8246 children with positive RT-PCR results were identified. After exclusions, 5740 were included: 1540 with SARS-CoV-2, 2150 with RSV, and 2050 with influenza. Comparison of included versus excluded patients revealed no clinically meaningful differences in age, sex distribution, or hospitalization rates (all SMD < 0.10). Baseline characteristics are summarized in Table 1.

### 3.2. Age-stratified post-acute symptom prevalence

Because the 3 viral cohorts differed substantially in age distribution, age-stratified prevalence is presented here as the primary comparison, ahead of the per-virus results below; overall (age-unadjusted) prevalence figures are confounded by the differing age structure of each cohort. Stratified analyses by age group (infant [<12 months], preschool [1–6 years], and school-age [>6 years]) confirmed that the RSV-dominant respiratory pattern was most pronounced in infants, while the SARS-CoV-2 neurocognitive pattern was strongest in school-aged children. Importantly, age-stratified post-acute symptom prevalence revealed distinct patterns: among infants <12 months, RSV prevalence was 28.0% compared with 16.2% for SARS-CoV-2 and 18.1% for influenza; among school-aged children >6 years, SARS-CoV-2 prevalence was 26.0% compared with 23.4% for influenza and 0.0% for RSV (no persistent symptoms were documented in this small subgroup, n = 107). Formal testing for effect modification using virus × age group interaction terms in logistic regression models confirmed significant interaction (*P* < .001), indicating that the relationship between viral etiology and post-acute symptom risk is age-dependent (Table 2).

**Table 2:** Age-stratified post-acute symptom prevalence by viral etiology.

Age group	SARS-CoV-2, n/N (%, 95% CI)	RSV, n/N (%, 95% CI)	Influenza, n/N (%, 95% CI)
<6 mo	10/92 (10.9%, 6.0–18.9)	248/1204 (20.6%, 18.4–23.0)	23/164 (14.0%, 9.5–20.2)
6–12 mo	27/139 (19.4%, 13.7–26.8)	264/624 (42.3%, 38.5–46.2)	44/205 (21.5%, 16.4–27.6)
13 mo–6 yr	110/570 (19.3%, 16.3–22.7)	4/215 (1.9%, 0.7–4.7)	147/738 (19.9%, 17.2–23.0)
>6 yr	192/739 (26.0%, 22.9–29.3)	0/107 (0.0%, 0.0–3.5)	221/943 (23.4%, 20.8–26.2)
Total	339/1540 (22.0%)	516/2150 (24.0%)	435/2050 (21.2%)

Data are n/N (%) with Wilson 95% CIs. Persistent symptoms defined as the presence of any post-acute symptom at 4 to 6 weeks.

CI = confidence interval, RSV = respiratory syncytial virus, SARS-CoV-2 = severe acute respiratory syndrome coronavirus 2.

*P* value from chi-square test comparing prevalence across the 3 viral groups within each age stratum: <6 months, *P* = .015; 6–12 months, *P* < .001; 13 months–6 years, *P* < .001; and >6 years, *P* < .001. Formal testing for effect modification using virus × age group interaction terms confirmed a significant interaction (*P* < .001).

### 3.3. Severe acute respiratory syndrome coronavirus 2

At 4 to 6 weeks, 22.0% (95% CI = 19.9–24.1; n = 339/1540) of children with SARS-CoV-2 infection exhibited at least 1 persistent symptom. The dominant complaints were fatigue (156/339, 46%), sleep disturbance (129/339, 38%), headache (115/339, 34%), and concentration difficulties (98/339, 29%). Respiratory symptoms were relatively uncommon (persistent cough 11%, wheezing 5%). The neurocognitive symptom profile was predominantly observed in school-aged children (>6 years).

In multivariable analysis, age >6 years (aOR = 2.1, 95% CI = 1.5–2.9), hospitalization (aOR = 1.9, 95% CI = 1.4–2.6), and elevated inflammatory markers (aOR = 1.7, 95% CI = 1.2–2.4) were independently associated with post-acute symptom persistence. The model demonstrated adequate calibration (Hosmer-Lemeshow, *P* = .42) and acceptable discrimination (AUC = 0.71, 95% CI = 0.68–0.74).

### 3.4. Respiratory syncytial virus

Persistent symptoms were observed in 24.0% (95% CI = 22.2–25.8; n = 516/2150) of children with RSV infection. The cohort consisted predominantly of young infants (56% aged <6 months). Persistent symptoms were mainly respiratory and feeding-related: persistent cough (299/516, 58%), wheezing (243/516, 47%), feeding difficulties (227/516, 44%), and increased work of breathing (170/516, 33%). Neurocognitive symptoms were rare (<5%); however, this low frequency should be interpreted with caution, as headache, concentration difficulties, and sleep disturbance require verbal self-report and were only assessable in children aged ≥2 to 3 years, who constituted a minority of the RSV cohort.

In multivariable analysis, hypoxemia (aOR = 2.4, 95% CI = 1.7–3.3), hospitalization (aOR = 2.0, 95% CI = 1.4–2.8), age <6 months (aOR = 1.9, 95% CI = 1.4–2.6), and prematurity (aOR = 1.7, 95% CI = 1.2–2.4) were independently associated with post-acute symptoms. Palivizumab prophylaxis demonstrated a protective association (aOR = 0.6, 95% CI = 0.4–0.9). Model calibration was adequate (Hosmer-Lemeshow, *P* = .38) with good discrimination (AUC = 0.74, 95% CI = 0.71–0.77).

### 3.5. Influenza

Persistent symptoms at 4 to 6 weeks were documented in 21.2% (95% CI = 19.4–23.0; n = 435/2050). The most common symptoms were fatigue (178/435, 41%), myalgia (165/435, 38%), and prolonged fever (122/435, 28%). Headache (104/435, 24%) and reduced exercise tolerance (74/435, 17%) were also reported.

In multivariable analysis, prolonged fever (aOR = 2.0, 95% CI = 1.5–2.7), acute-phase fatigue/myalgia (aOR = 1.8, 95% CI = 1.3–2.4), and hospitalization (aOR = 1.7, 95% CI = 1.2–2.3) were independently associated with symptom persistence. Model calibration was satisfactory (Hosmer-Lemeshow, *P* = .51) with acceptable discrimination (AUC = 0.68, 95% CI = 0.64–0.72). Independent predictors of symptom persistence for each viral etiology are summarized in Table 3.

**Table 3:** Independent predictors of symptom persistence by viral etiology (multivariable logistic regression).

Predictor	aOR (95% CI)	*P* value	AUC (95% CI)
SARS-CoV-2 (n = 1309)			0.71 (0.68–0.74)
Age >6 yr	2.1 (1.5–2.9)	<.001	
Hospitalization	1.9 (1.4–2.6)	<.001	
Elevated inflammatory markers	1.7 (1.2–2.4)	.002	
RSV (n = 1892)			0.74 (0.71–0.77)
Hypoxemia (SpO_2_ < 92%)	2.4 (1.7–3.3)	<.001	
Hospitalization	2.0 (1.4–2.8)	<.001	
Age <6 mo	1.9 (1.4–2.6)	<.001	
Prematurity (<37 wk)	1.7 (1.2–2.4)	<.001	
Palivizumab prophylaxis*	0.6 (0.4–0.9)	.014	
Influenza (n = 1783)			0.68 (0.64–0.72)
Prolonged fever (>7 d)	2.0 (1.5–2.7)	<.001	
Acute-phase fatigue/myalgia	1.8 (1.3–2.4)	<.001	
Hospitalization	1.7 (1.2–2.3)	.002	

All models adjusted for age, sex, and hospitalization status.

Hosmer-Lemeshow goodness-of-fit test *P* > .05 for all 3 models.

VIF < 2.5 for all predictors.

aOR = adjusted odds ratio, AUC = area under the receiver operating characteristic curve (C-statistic), CI = confidence interval, RSV = respiratory syncytial virus, SARS-CoV-2 = severe acute respiratory syndrome coronavirus 2, SpO_2_ = peripheral oxygen saturation, VIF = variance inflation factor.

* Protective association (OR < 1.0).

**Figure 1.**
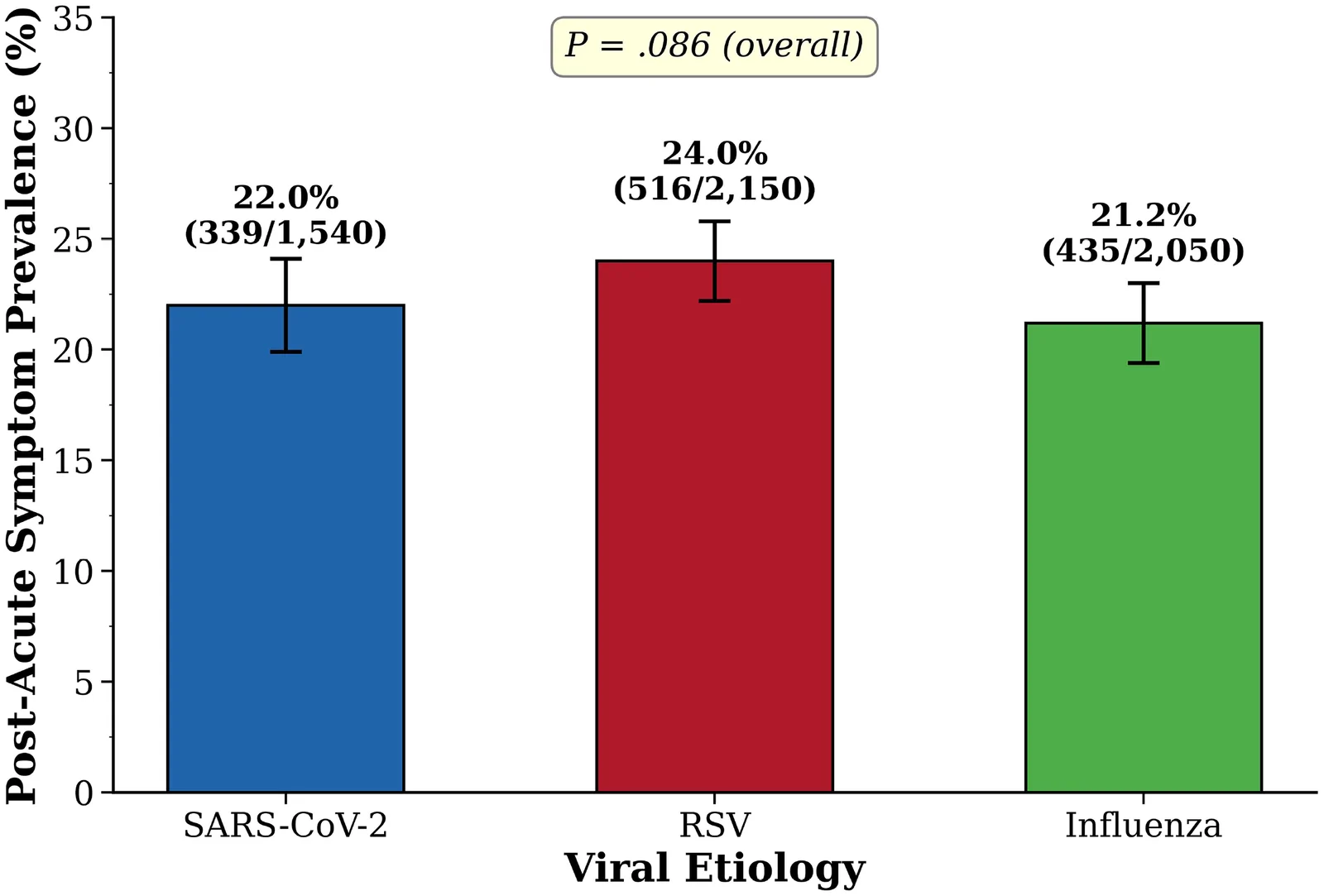
Post-acute symptom prevalence at 4 to 6 weeks by viral etiology. Prevalence defined as the proportion of children with at least 1 persistent symptom at clinician-documented follow-up. *P* value from chi-square test. SARS-CoV-2: 339/1540 (22.0%); RSV: 516/2150 (24.0%); Influenza: 435/2050 (21.2%). RSV = respiratory syncytial virus, SARS-CoV-2 = severe acute respiratory syndrome coronavirus 2.

**Figure 2.**
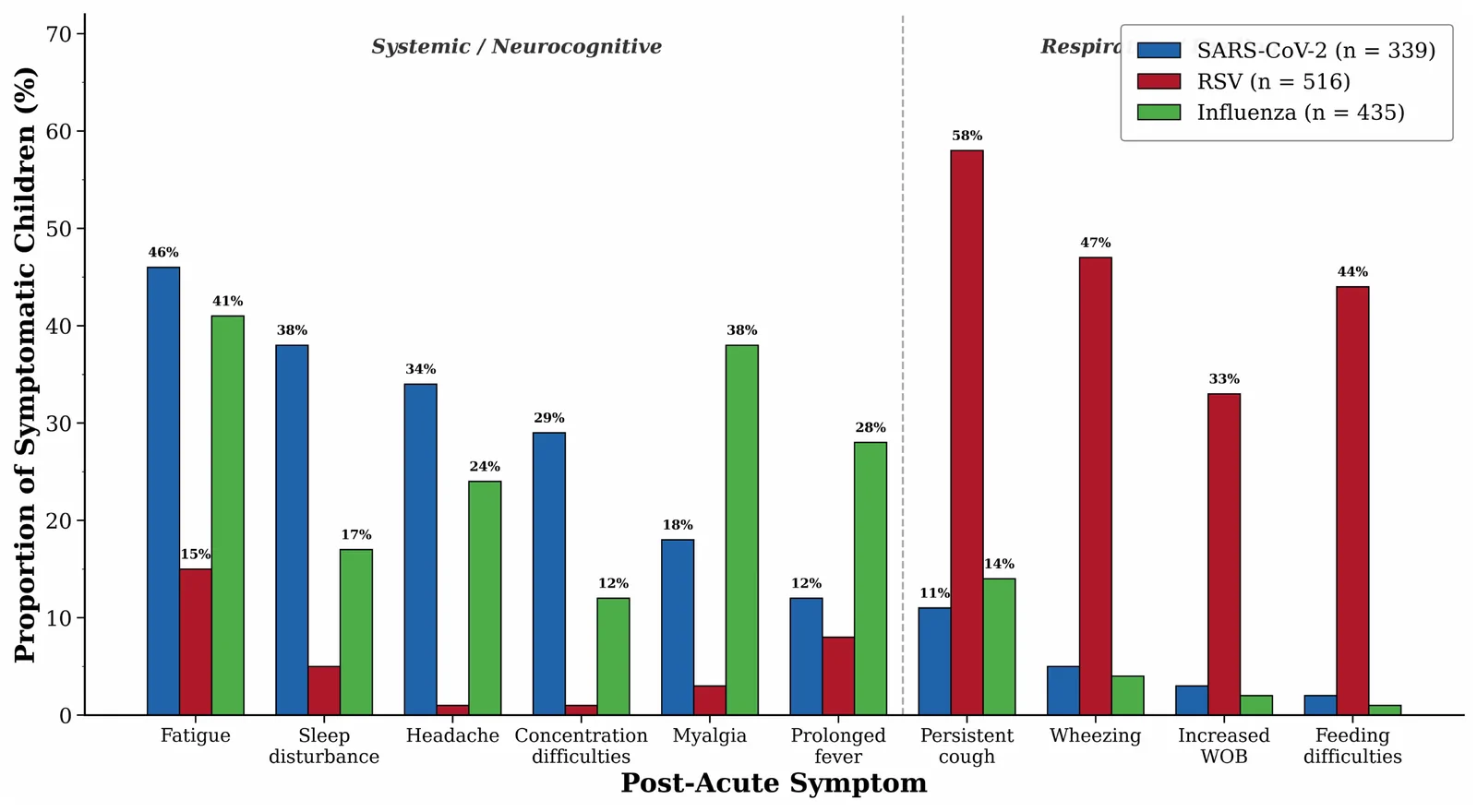
Distribution of post-acute symptoms at 4 to 6 weeks by viral etiology. Percentages are calculated within the subgroup of children with documented persistent symptoms in each viral group (SARS-CoV-2: 339/1540; RSV: 516/2150; Influenza: 435/2050). Symptoms are grouped by domain: systemic/neurocognitive (left) and respiratory/feeding (right). RSV = respiratory syncytial virus, SARS-CoV-2 = severe acute respiratory syndrome coronavirus 2, WOB = work of breathing.

Overall post-acute symptom prevalence by viral etiology is presented in Figure 1. Symptom domain distribution is summarized in Figure 2 and Table 4.

**Table 4:** Persistent symptoms at 4 to 6 weeks by viral etiology.

Symptom	SARS-CoV-2, n/N (%)	RSV, n/N (%)	Influenza, n/N (%)
Systemic/neurocognitive			
Fatigue*	156/339 (46%)	77/516 (15%)	178/435 (41%)
Sleep disturbance	129/339 (38%)	26/516 (5%)	74/435 (17%)
Headache†	115/339 (34%)	5/516 (1%)	104/435 (24%)
Concentration difficulties†	98/339 (29%)	5/516 (1%)	52/435 (12%)
Myalgia†	61/339 (18%)	15/516 (3%)	165/435 (38%)
Prolonged fever‡	41/339 (12%)	41/516 (8%)	122/435 (28%)
Respiratory/feeding			
Persistent cough§	37/339 (11%)	299/516 (58%)	61/435 (14%)
Wheezing§	17/339 (5%)	243/516 (47%)	17/435 (4%)
Increased work of breathing§	10/339 (3%)	170/516 (33%)	9/435 (2%)
Feeding difficulties‖	7/339 (2%)	227/516 (44%)	4/435 (1%)

Percentages calculated among children with documented persistent symptoms within each viral group.

RSV = respiratory syncytial virus, SARS-CoV-2 = severe acute respiratory syndrome coronavirus 2.

* Infants: reduced activity, decreased alertness, or prolonged sleep beyond pre-illness baseline.

† Assessed in verbal children aged ≥2 to 3 years.

‡ Axillary temperature ≥38.0°C persisting or recurring beyond day 7.

§ Clinician-documented on auscultation or clinical examination.

‖ Oral intake <50% of pre-illness baseline or modified feeding required.

### 3.6. Sensitivity and subgroup analyses

Among hospitalized versus outpatient-managed children, hospitalization was consistently associated with higher post-acute symptom prevalence across all 3 viral groups (28.8% vs 18.6% overall, *P* < .001). IPW analysis yielded prevalence estimates within 1.5 percentage points of the primary complete-case analysis for all 3 viruses, supporting robustness to potential selection bias. Temporal analysis comparing pandemic-era (2020–2021) versus post-pandemic (2022–2024) SARS-CoV-2 infections showed no statistically significant difference in overall post-acute prevalence (23.5% vs 20.8%, *P* = .21), though post-pandemic infections trended toward lower neurocognitive symptom burden. In a post hoc, unadjusted analysis of vaccination status (available for a subset of the SARS-CoV-2 and influenza cohorts; missing data 6.8%–7.4%), COVID-19 vaccination showed no significant association with post-acute symptom persistence (crude OR = 1.17, 95% CI = 0.77–1.78), whereas influenza vaccination was associated with significantly lower odds of persistent symptoms (crude OR = 0.51, 95% CI = 0.39–0.67).

## 4. Discussion

This real-world cohort study of 5740 children demonstrates that post-acute symptom persistence at 4 to 6 weeks following pediatric viral respiratory infections follows distinct, virus-specific clinical patterns. Post-acute symptom prevalence was comparable across RSV (24.0%), SARS-CoV-2 (22.0%), and influenza (21.2%; overall *P* = .086), indicating that all 3 major pediatric respiratory viruses produce clinically meaningful post-acute morbidity at similar rates. However, the composition of persistent symptoms differed markedly across viral etiologies. These findings suggest that post-acute infectious morbidity in children is not confined to SARS-CoV-2 and that other major respiratory viruses may warrant comparable clinical attention during the post-acute recovery period. The 4- to 6-week interval reflects standardized institutional follow-up practice and was selected to capture early post-acute morbidity while minimizing recall variability; we acknowledge that this time point captures a snapshot of the post-acute course rather than defining definitive long-term outcomes.

An important interpretive caveat is warranted: the 3 viral cohorts differed substantially in age distribution (RSV median 10.5 months vs SARS-CoV-2 median 7.4 years vs influenza median 7.2 years), and the overall prevalence comparisons are therefore partially confounded by age. Age-stratified analyses demonstrated a significant virus × age group interaction (*P* < .001), with RSV prevalence highest among infants (28.0%) and SARS-CoV-2 prevalence highest among school-aged children (26.0%). Accordingly, the headline prevalence differences should be interpreted as reflecting the combined effect of viral etiology and the age-specific populations each virus predominantly affects, rather than a purely virus-attributable difference. This age-dependency is itself clinically informative, as it identifies which patient subgroups are at greatest risk following each specific infection.

The SARS-CoV-2 post-acute prevalence of 22.0% is consistent with the range reported in pediatric long COVID literature. A meta-analysis of 40 studies estimated a pooled prevalence of 23.4% (95% CI = 15.8%–32.5%),^[4–6]^ and the RECOVER initiative reported cumulative long COVID incidence of 15% to 20% across pediatric age groups.^[10]^ Our estimate aligns closely with these figures, supporting the external validity of the present cohort. The predominance of fatigue, sleep disturbance, and concentration difficulties in the school-aged subgroup is consistent with multi-national cohort data.^[5,8,9,22]^

RSV-related post-acute morbidity was dominated by persistent respiratory and feeding difficulties in young infants, reflecting the prolonged time required for repair of respiratory epithelial damage following RSV bronchiolitis. RSV exhibits a distinct tropism for small airway epithelium, inducing extensive ciliary dysfunction, mucus plugging, and peribronchiolar inflammation that may require weeks to months for complete resolution.^[15]^ Furthermore, the immature immune response in young infants – characterized by a Th2-polarized cytokine environment and limited interferon-gamma production – may prolong the inflammatory resolution phase, contributing to sustained respiratory symptoms.^[16]^ The 24.0% prevalence is consistent with the lower range of emerging post-bronchiolitis follow-up data reporting persistent respiratory symptoms in 20% to 40% of hospitalized infants at 4 to 8 weeks.^[13,14,23]^ The protective association of palivizumab prophylaxis should be interpreted cautiously given the small subgroup size (n = 65) and wide CI (aOR = 0.6, 95% CI = 0.4–0.9); this exploratory finding is hypothesis-generating and aligns with the biological rationale that attenuating initial viral injury may reduce downstream post-acute morbidity, but requires confirmation in adequately powered prospective studies.

Influenza demonstrated the lowest post-acute symptom burden, with sustained systemic symptoms predominating, consistent with prior studies describing self-limited but sometimes prolonged functional recovery periods.^[17,18]^ The prominence of myalgia and reduced exercise tolerance suggests that influenza-related post-acute morbidity may reflect systemic inflammatory resolution dynamics rather than organ-specific viral damage.

### 4.1. Comparative perspective

The observation that all 3 viruses produced comparable overall post-acute symptom rates is noteworthy. While pediatric long COVID has received considerable attention, these data suggest that RSV and influenza produce post-acute morbidity at similar frequencies, though with fundamentally different symptom compositions. RSV-related post-acute respiratory morbidity has been comparatively underrecognized.^[24]^ A recent retrospective cohort study by Wee and colleagues compared long-term sequelae following hospitalization for RSV, Omicron-era SARS-CoV-2, and influenza and similarly identified an elevated respiratory sequelae burden after RSV. However, that study included both adults and children, was restricted to hospitalized patients, and relied on claims-based longer-term outcomes, whereas our pediatric-only cohort included both outpatient-managed and hospitalized children and evaluated clinician-documented symptoms at 4 to 6 weeks. These methodological differences limit direct numerical comparison but support the broader observation that post-RSV morbidity may extend beyond the acute phase across care settings and age groups. This is particularly relevant as RSV immunoprophylaxis expands with the introduction of nirsevimab and maternal RSV vaccination, which may reduce not only acute disease burden but also post-acute morbidity. Our exploratory finding of a protective palivizumab association, though limited by small sample size and wide CIs, generates a testable hypothesis for prospective evaluation in larger immunoprophylaxis cohorts.

### 4.2. What this study adds

To our knowledge, this represents one of the largest pediatric comparative cohorts to date directly comparing post-acute symptom profiles across SARS-CoV-2, RSV, and influenza within a unified clinical framework. Our study demonstrates that: post-acute symptom persistence occurs at comparable frequencies across all 3 viruses (21%–24%), indicating this is not unique to SARS-CoV-2; symptom profiles are virus-specific and age-dependent; identifiable acute-phase predictors can enable risk stratification at the time of initial infection; and RSV-related post-acute morbidity may warrant greater clinical attention than currently allocated in routine pediatric practice.

### 4.3. Clinical implications

We do not suggest that our findings introduce an entirely novel clinical concept; post-discharge follow-up is generally guided by clinical presentation and severity. Rather, these data provide structured evidence for what may already be intuitive practice and support the development of virus-specific, risk-stratified follow-up protocols. Specifically, infants with RSV bronchiolitis complicated by hypoxemia, prematurity, or young age (<6 months) may benefit from scheduled respiratory reassessment at 4 to 6 weeks; school-aged children recovering from SARS-CoV-2 with elevated inflammatory markers may warrant neurocognitive screening; and children with influenza complicated by prolonged fever may benefit from functional recovery monitoring.

### 4.4. Generalizability

This study was conducted at a single university hospital system in Istanbul, Turkey. While the institutional setting provides methodological advantages – uniform diagnostic protocols, standardized follow-up practices, and consistent EMR documentation – the generalizability to other populations deserves consideration. Turkey’s pediatric demographic profile, with a relatively young population and moderate vaccination coverage during the study period, may influence both acute infection epidemiology and post-acute outcomes. The tertiary-care referral setting may overrepresent more severe infections compared to primary care populations. However, the inclusion of both hospitalized and outpatient-managed patients across a broad age range (0–18 years), the 5-year study period encompassing multiple respiratory seasons, and consistent results across sensitivity analyses support the broader applicability of the observed virus-specific patterns, even if absolute prevalence estimates may differ in other settings.

### 4.5. Limitations

Several limitations should be considered. First, the retrospective design and reliance on clinician-documented symptoms introduce ascertainment variability; clinicians may not have systematically queried all symptom domains at every follow-up visit, potentially underestimating milder symptoms. The absence of standardized symptom questionnaires is inherent to the retrospective design. Importantly, follow-up visits were scheduled according to institutional post-discharge and outpatient protocols rather than symptom-triggered self-selection alone, which mitigates but does not eliminate potential ascertainment bias. Second, preexisting conditions not meeting exclusion criteria (e.g., mild atopic disease) may have influenced symptom reporting. Third, differential symptom ascertainment across age groups represents an important source of measurement bias: neurocognitive symptoms (headache, concentration difficulties) require verbal self-report and were only assessable in children aged ≥2 to 3 years, systematically underestimating these complaints in the predominantly infant RSV cohort. Cross-viral symptom profile comparisons should therefore be interpreted as reflecting both true biological differences and age-related assessment limitations. Fourth, the 4- to 6-week assessment window was selected to capture early post-acute morbidity at a clinically actionable time point while minimizing recall bias and aligning with standardized institutional follow-up scheduling; however, this window does not address longer-term trajectories, and future studies with serial assessments at 3, 6, and 12 months are needed to characterize symptom evolution and resolution dynamics. Fifth, the requirement for a documented follow-up visit introduces a form of immortal time bias: children who did not receive a documented follow-up visit (52% of all exclusions) may differ systematically from those included by acute illness severity and clinical trajectory in ways not captured by our SMD comparison of measured baseline characteristics. This source of bias cannot be fully quantified with the available data; the SMD and IPW sensitivity analyses provide partial reassurance limited to the measured covariates and do not eliminate this concern. Sixth, selection bias cannot be entirely excluded, though our comparison of included versus excluded patients showed no meaningful baseline differences (all SMD < 0.10) and IPW sensitivity analysis yielded prevalence estimates within 1.5 percentage points of primary results, supporting robustness. Seventh, temporal confounding related to evolving SARS-CoV-2 variants, vaccination coverage changes, and seasonal variation may have influenced results, though subgroup temporal analysis did not reveal significant period effects. Eighth, symptoms were coded as binary (present/absent) without severity grading; future prospective studies incorporating validated symptom severity scales (e.g., PedsQL, International Severe Acute Respiratory and Emerging Infection Consortium pediatric follow-up instruments) would provide more granular characterization. Ninth, the post hoc vaccination status analysis was unadjusted for confounders such as age, underlying health status, and healthcare-seeking behavior, and residual confounding by indication cannot be excluded; for RSV, immunoprophylaxis data were limited to palivizumab (n = 65), and nirsevimab and maternal RSV vaccination data were not available in this cohort. Finally, the single-center design limits geographic generalizability, as discussed above.

## 5. Conclusion

Persistent symptoms at 4 to 6 weeks following pediatric viral respiratory infections are common and follow virus-specific patterns. In this hospital-based cohort, all 3 major respiratory viruses produced comparable overall post-acute symptom prevalence (range, 21.2%–24.0%), but with distinct, virus-specific symptom domain profiles and independent predictors for each pathogen. These findings support the need for virus-specific, risk-stratified follow-up approaches in pediatric clinical practice and suggest that post-acute infectious morbidity in children extends beyond SARS-CoV-2. Prospective multicenter studies using standardized, age-appropriate outcome instruments are needed to validate these findings and determine whether targeted, pathogen-informed follow-up improves functional recovery.

## Acknowledgments

The authors acknowledge the biostatistical consultation provided by the Department of Biostatistics, Istanbul Medipol University.

## Author contributions

**Conceptualization:** Firat Bedir.

**Data Curation:** Firat Bedir.

**Formal Analysis:** Firat Bedir.

**Methodology:** Firat Bedir.

**Resources:** Firat Bedir.

**Software:** Firat Bedir.

**Supervision:** Velit Demir.

**Validation:** Velit Demir.

**Writing – Original Draft:** Firat Bedir.

**Writing – Review & Editing:** Velit Demir.
